# Extracellular Vesicle-Mediated miR-155 from Visceral Adipocytes Induces Skeletal Muscle Dysplasia in Obesity

**DOI:** 10.3390/cells14171302

**Published:** 2025-08-22

**Authors:** Yunyan Ji, Zeen Gong, Rui Liang, Di Wu, Wen Sun, Xiaomao Luo, Yi Yan, Jiayin Lu, Juan Wang, Haidong Wang

**Affiliations:** 1 College of Veterinary Medicine, Shanxi Agricultural University, Jinzhong 030801, China; 18306873350@163.com (Y.J.); 18404983660@163.com (Z.G.); 15513918599@163.com (R.L.); fkx3853946@163.com (D.W.); lionelphilip@126.com (X.L.); yanyi@sxau.edu.cn (Y.Y.); jiayinlu@sxau.edu.cn (J.L.); 2 Department of Nephrology, Shanghai General Hospital, Shanghai Jiao Tong University School of Medicine, Shanghai 200080, China; sunwensj@sjtu.edu.cn (W.S.); wangdanwj@126.com (J.W.)

**Keywords:** obesity, extracellular vesicle, miR-155, macrophage, skeletal muscle

## Abstract

Obesity poses a serious threat to human health, with induced skeletal muscle dysfunction significantly increasing the risk of metabolic syndrome. In obesity, it is known that visceral adipose tissue (VAT) mediates the dysregulation of the adipose–muscle axis through exosome-delivered miRNAs, but the associated regulatory mechanisms remain incompletely elucidated. This study established an AAV-mediated miR-155 obese mouse model and a co-culture system (HFD VAD-evs/RAW264.7 cells/C2C12 cells) to demonstrate that high-fat diet-induced VA-derived extracellular vesicles (HFD VAD-evs) preferentially accumulate in skeletal muscle and induce developmental impairment. HFD VAD-evs disrupt skeletal muscle homeostasis through dual mechanisms: the direct suppression of myoblast development via exosomal miR-155 cargo and the indirect inhibition of myogenesis through macrophage-mediated inflammatory responses in skeletal muscle. Notably, miR-155 inhibition in HFD VAD-evs reversed obesity-associated myogenic deficits. These findings provide novel mechanistic insights into obesity-induced skeletal muscle dysregulation and facilitate potential therapeutic strategies targeting exosomal miRNA signaling.

## 1. Introduction

As a modifiable risk factor, obesity continues to manifest as a principal contributor to the disease burden worldwide, with global prevalence rates increasing by 155.1% in males and 104.9% in females between 1990 and 2021 [1]. The chronic consumption of a high-fat diet (HFD) drives obesity development, leading to the pathological accumulation of visceral adipose tissue (VAT) [2,3]. Obesity-associated skeletal muscle dysfunction manifests through localized pro-inflammatory responses [4,5], muscle atrophy [6,7,8], and reduced protein synthesis [9], ultimately impairing muscle functionality.

Leptin, adiponectin, and resistin—signaling molecules secreted by adipose tissue—play pivotal roles in modulating metabolic homeostasis, inflammatory responses, and insulin signaling pathways [10,11,12,13]. Notably, in obese individuals, inflamed VAT promotes systemic low-grade inflammation [14,15]. Exosomes, a novel class of adipokines [16], mediate intercellular communication by transferring signals from visceral adipocytes, modulating insulin resistance, glucose intolerance [17], and skeletal muscle development [18]. Obese individuals exhibit elevated levels of circulating exosomal miRNAs, predominantly derived from adipose tissue [19,20], which regulate skeletal and cardiac muscle function [21]. Notably, miR-155 is highly expressed in obese adipose tissue, exacerbating adipocyte inflammation [22], and is abundantly present in the peripheral blood of type 2 diabetes patients [23]. The mechanistic insights from these studies establish miR-155 as a promising molecular candidate for intervening in obesity-related metabolic dysregulation. However, the mechanism by which VAD-derived exosomal miR-155 regulates skeletal muscle homeostasis remains unclear.

Macrophages are critical regulators of the physiology and pathological equilibrium of skeletal muscle [24], and pathological contexts drive macrophage polarization toward an IL-1β/TNF-α-secreting M1 or an IL-10/TGF-β-producing M2 phenotype, creating therapeutic opportunities for immune modulation [25,26]. The immunometabolic microenvironment of lean adipose depots exhibits preferential accumulation of M2-polarized macrophages, which maintain metabolic homeostasis [25]. In obesity, macrophages shift to an M1 phenotype, secreting pro-inflammatory cytokines that amplify local inflammation [25,26], while adipose tissue expansion recruits and activates immune cells, initiating a cascade of inflammatory responses [27]. During muscle injury repair, macrophages dynamically modulate their polarization states to clear necrotic debris, initiate tissue regeneration, and regulate inflammatory progression [28]. Emerging evidence highlights macrophages as key mediators of adipose–muscle crosstalk [29], and adipose tissue macrophages can secrete exosomes containing miR-155 that are delivered to the muscle, liver, and adipocytes [30], though the underlying mechanisms require further elucidation.

This study demonstrates that obesity induces skeletal muscle dysplasia in mice through dual mechanisms mediated by HFD VAD-evs carrying miR-155. First, these HFD VAD-evs directly suppress the development of skeletal myoblasts via their exosomal miR-155 cargo, and second, this miR-155 promotes the pro-inflammatory polarization of skeletal muscle macrophages, which further inhibits skeletal myoblasts’ development. Critically, the inhibition of miR-155 in HFD VAD-evs reversed obesity-associated skeletal muscle developmental deficits, highlighting the central role of exosomal miR-155 in driving muscle pathologies. These findings provide mechanistic insights into the adipose–muscle crosstalk in obese individuals and identify exosomal miR-155 as a therapeutic target for metabolic disorders.

## 2. Materials and Methods

### 2.1. Experimental Animals

Male C57BL/6 mice (4 weeks old or 6 weeks old) and male balb/c nude mice (6 weeks old) were purchased from SPF Biotechnology Co., Ltd. (Beijing, China). All the experimental procedures were conducted in accordance with guidelines approved by the Institutional Animal Care and Use Committee of Shanxi Agricultural University (Protocol No. SXAU-EAW-2023M.SW.011009273). Obesity was confirmed with the observation of significant body weight gain.

Following a 7-day acclimation period under specific pathogen-free (SPF) conditions, the 6-week-old mice were randomly divided into 3 groups (*n* = 3/group) and were fed a normal diet. The specific handling processes are shown in Table 1.

After the 4-week-old mice were adaptively fed for one week, they were divided into 5 groups (*n* = 3/group) at random and received an HFD. The specific handling processes are shown in Table 2.

### 2.2. Oral Glucose Tolerance Test (OGTT) and Insulin Tolerance Test (ITT)

The OGTT was conducted following a 12-h overnight fast. Mice received an oral gavage of glucose solution (2 g/kg body weight in water) at 0 min, blood samples were collected via sterile tail-vein puncture, and blood glucose was measured every 30 min for 120 min using a glucometer (Roche, Indianapolis, IN, USA).

The ITT was conducted following a 12-h overnight fast. Mice were injected intraperitoneally with insulin (0.5 IU/kg body weight), blood samples were collected, and blood glucose was measured after 0, 15, 30, 60, and 120 min.

### 2.3. Body Composition Measurement and Tissue Collection

Body composition analysis was performed using a Bruker Minispec LF50 analyzer (Bruker, Rheinstetten, Germany). Mice were intraperitoneally anesthetized using 3% sodium pentobarbital, and blood was collected to isolate plasma, which was stored at −80 °C. Following euthanasia, subcutaneous adipose tissue, visceral adipose tissue (perirenal fat pads and epididymal fat pads), and gastrocnemius muscle were harvested. To assess the relative size of organs, the organ coefficient was calculated as Organ coefficient = (Organ weight/Body weight) × 100 (g/100 g), and it was used for statistical analysis.

### 2.4. Histological Analysis

Skeletal muscle and adipose tissue samples underwent 4% paraformaldehyde fixation (24–48 h) followed by hematoxylin–eosin (H&E) staining of paraffin-embedded sections [31]. Whole-slide images were acquired for subsequent analysis.

Control or experimental co-cultures were fixed in cold methanol at 4 °C for 10 min. Subsequently, cells were stained with the Giemsa and May-Grünwald dyes (Sigma, Budapest, Hungary) according to the manufacturer’s protocol and examined under an Eclipse TE 200 microscope (Nikon, Yokohama, Japan) to determine the cell fusion index.

### 2.5. Western Blot

Before being transferred onto PVDF membranes, protein samples (20 µg) were separated on SDS-PAGE gels. Then, the membranes were blocked with 5% skim milk for 1 h and incubated with primary antibodies at 4 °C overnight. The next day, membranes were incubated with HRP-conjugated secondary antibodies for 1 h at room temperature. Protein expression levels were normalized to α-tubulin as a control.

### 2.6. Enzyme-Linked Immunosorbent Assay (ELISA)

Skeletal muscle tissues were homogenized, and the supernatant was collected (14,000× *g*, 15 min). Protein levels of the supernatant and plasma were measured using a BCA assay kit (Beyotime, Suzhou, China), along with commercial ELISA kits containing anti-IL-1β (Meimian, Shanghai, China) and anti-IL-6 (Meimian, #4355) antibodies.

### 2.7. Cell Culture

Mouse C2C12 myoblasts and RAW264.7 macrophages were cultured in Dulbecco’s modified Eagle’s medium (DMEM) supplemented with 10% heat-inactivated fetal calf serum (FCS), 200 U/mL penicillin, and 100 µg/mL streptomycin at 37 °C under 5% CO_2_. To induce differentiation, once cells had achieved >95% confluency, the growth medium was replaced with low-serum differentiation medium (DMEM containing 2% horse serum; Solarbio, Beijing, China) for a 3-day culture period.

### 2.8. Isolation of Single Cells from Skeletal Muscles

The isolation and culturing of skeletal muscle cells were carried out as described previously [32,33]. Briefly, skeletal muscle tissue (50 mg) from mice was minced as finely as possible, and then was incubated for half an hour with Collagenase II (125 CUD/mg, Sigma, Budapest, Hungary). Then, this tissue was digested using a mixture of CollageD (0.24 U/mg, RD, #11088858001)/disease II (1.1 U/mg, Sigma, #D4693), 2% bovine serum albumin (Sigma, #9048-46-8), and 150 μM CaCl_2_. The digested tissue was sequentially filtered through 100 μm and 40 μm strainers and centrifuged to isolate skeletal muscle cells.

### 2.9. Flow Cytometry

Single-cell suspensions of skeletal muscle tissue were incubated with antibodies (F4/80-Alexa Fluor 488, CD206-PE, CD11b-APC and CD86-PE. BioLegend, San Diego, CA, USA) for 1 h, followed by incubation with the viability dye eFluor™ 780 (Thermo Fisher Scientific, Waltham, MA, USA) for 15 min. Cells were then washed twice with cold PBS. Flow cytometry was performed by means of an FACS Calibur flow cytometer (Meimian, Shanghai, China) and data were analyzed using FlowJo 10.0.7 software (Tree Star, Ashland, OR, USA).

### 2.10. Plasma Extracellular Vesicle Isolation

An exosome isolation kit (Umibio, Shanghai, China) was used to isolate plasma extracellular vesicles (plasma-evs). In brief, plasma-evs were extracted from equal volumes of plasma for each sample, with cellular debris being removed (10,000× *g*, 1 h). Subsequently, 500 μL of the supernatant was mixed with 400 μL of exosome precipitation solution A to deplete plasma proteins, followed by the addition of 120 μL of solution B. The mixture was centrifuged at 12,000 rpm for 15 min and further purified using an exosome purification filter at 5600 rpm for 20 min. The final exosome pellet was resuspended in 50 μL of PBS.

### 2.11. Isolation, Culture, and Exosome Collection of Visceral Adipocytes

The isolation and culture of visceral adipocytes were performed as previously described [34,35,36]. Briefly, visceral adipose tissue (50 mg) from mice was minced as finely as possible and digested using a mixture composed of enzyme collagenase type II (2 mg/mL, Sigma, #C6885), 2% bovine serum albumin (Sigma, #9048-46-8), and 150 μM CaCl_2_. The digested tissue was sequentially filtered through 100 μm and 40 μm strainers, red blood cell lysis buffer (Beyotime, Beijing, China) was added, and centrifugation was performed to isolate adipocytes.

Visceral adipocytes were cultured in serum-free medium for 24 h until minimal cell death was observed. The supernatant was collected and sequentially centrifuged at 300× *g* for 10 min, 2000× *g* for 10 min, and 10,000× *g* for 30 min. The clarified supernatant was filtered through a 0.22 μm filter and subjected to exosome isolation via centrifugation at 100,000× *g* for 1.5 h, repeated twice. Purified extracellular vesicles were resuspended for subsequent experiments.

### 2.12. Ev Characterization

Nanoparticle tracking analysis (NTA) was performed using a ZetaView^®^ PMX 110 (Malvern Panalytical, Westborough, MA, USA). For morphological analysis, transmission electron microscopy (TEM) was conducted using a Tecnai G2 Polara microscope (Thermo Fisher Scientific, Waltham, MA, USA).

### 2.13. EV Labeling and Tracking

EVs (approximately 1 μg/μL protein content) were incubated with 1 mM DiI (Beyotime, Beijing, China) or DiR (Umibio, Shanghai, China) at 500:1 for 30 min. Free dye was removed by means of centrifugation at 100,000× *g* for 1.5 h. Nude mice received 100 μg of DiR-EVs via injection into the tail vein. After 24 h, whole-body and organ-specific exosome localization was detected using an IVIS Lumina II in vivo imaging system (Thermo Fisher Scientific, Waltham, MA, USA).

At 24 h post-intravenous injection of DiL-labeled EVs, skeletal muscle tissues were harvested and sectioned into frozen slices [37]. Tissue sections were blocked with 5% bovine serum albumin (BSA) for 1 h, followed by incubation with anti-F4/80 antibody (Abcam; Cambridge, UK) overnight. After washing three times, sections were incubated with Alexa Fluor 488-conjugated secondary antibody (Invitrogen, Carlsbad, CA, USA) for 1 h at 24 °C in the dark, and exosome localization in skeletal muscle was examined via fluorescence microscopy.

### 2.14. Cell Transfection

MiR-155 mimic and inhibitor were synthesized by GenePharma (GenePharma, Suzhou, China) and transfected into cells. Briefly, after cells were cultured to subconfluency, 7.5 μL of Lipofectamine 2000 reagent (Invitrogen, Carlsbad, CA, USA) and 120 pmol of miR-155 mimic or inhibitor were added to each well of a 6-well plate. After 6–8 h of transfection, cells were cultured in DMEM containing 10% fetal bovine serum to maintain proliferation or in a differentiation medium to induce myotube formation. RNA oligonucleotide sequences are provided in Table 3.

### 2.15. Co-Culture of HFD Visceral Adipocyte Cells with Myoblasts

C2C12 cells (1 × 10^5^ cells/well) were seeded in the lower chamber of Transwell plates, while HFD VA cells (1 × 10^5^ cells/well) were plated in the upper chamber (pore size 0.4 μm) and cultured in serum-free DMEM with (CO-HFD VA) or without GW4869 (CO-GW4869).

### 2.16. HFD VAD-evs/RAW264.7 Cells/C2C12 Cells Co-Culture System

Serum-free conditioned medium (SFCM) was collected from RAW264.7 macrophages. Additionally, SFCM was harvested from RAW264.7 cells treated for 24 h with either HFD VAD-evs loaded with the miR-155 inhibitor negative control (SFCM NCev) or HFD VAD-evs loaded with miR-155 inhibitor (SFCM INev). C2C12 cells were incubated with SFCM, SFCM NCev, or SFCM INev for 24 h to assess proliferation, or for 3 days to evaluate differentiation [38].

### 2.17. EDU Proliferation Assay

Cell proliferation was assessed using the BeyoClick™ EDU Cell Proliferation Kit with Alexa Fluor 594 (Beyotime, Beijing, China). Briefly, treated C2C12 cells (2 × 10^4^ cells/well) were seeded in 12-well plates, incubated with EDU for 2 h, fixed with 4% paraformaldehyde for 15 min, permeabilized with 0.3% Triton X-100 for 15 min, and incubated with Click reaction solution in the dark at room temperature for 30 min. Nuclei were counterstained with Hoechst 33342 for 10 min.

### 2.18. CCK-8 Assay

Cell suspensions (4 × 10^4^ cells/mL) were seeded into 96-well plates (100 μL/well). After 24 h, 10 μL of CCK-8 reagent was added to each well, and cells were cultured at 37 °C with 5% CO_2_ for 2 h, after which absorbance was obtained at 490 nm.

### 2.19. RNA Isolation and qRT-PCR

Total RNA was extracted using TRIzol reagent (Takara Bio, Shiga, Japan) following the manufacturer’s instructions. Approximately 2 μg of RNA was reverse-transcribed into cDNA using the miRcute Plus miRNA qPCR Detection Kit (Vazyme) or HiScript III RT SuperMix (Vazyme, Nanjing, China), and gene expression was analyzed with SYBR Green PCR Master Mix (Vazyme, Nanjing, China) on a quantitative real-time PCR system. The relative expression levels of miRNAs and mRNAs were calculated using the 2^−ΔΔCt^ method, and the primer sequences are listed in Table 3.

### 2.20. Forelimb Grip Strength Measurement

Forelimb grip strength was measured using a calibrated grip strength meter (Huateng, Guangzhou, China). All tests were performed as previously described [39], and grip strength values were recorded as the mean of at least three consecutive trials.

### 2.21. Loading of miRNA into EVs

The miR-155 inhibitor was loaded into HFD VAD-evs using the ExoLoad Kit (Echo-biotech, Beijing, China). Briefly, 100 μL of HFD VAD-evs (1 mg/mL) was mixed with 1000 pmol of miR-155 inhibitor and 200 μL of ETP solution, followed by incubation at 37 °C with shaking at 150 rpm for 2 h. Following incubation, EVs were concentrated to a final volume of 100 μL via ultrafiltration (100 kDa filter) and centrifugation at 4000× *g*, after which they were washed twice with washing buffer and collected for further use.

### 2.22. Statistical Analysis

All data were expressed as the mean ± SEM. Statistical significance between two groups was analyzed using Student’s t-test, while comparisons among more than three groups were assessed by ANOVA using GraphPad Prism 7.0 software. *p* < 0.05 was considered statistically significant.

## 3. Results

### 3.1. Skeletal Muscle Dysplasia in Obesity

**Figure 1:**
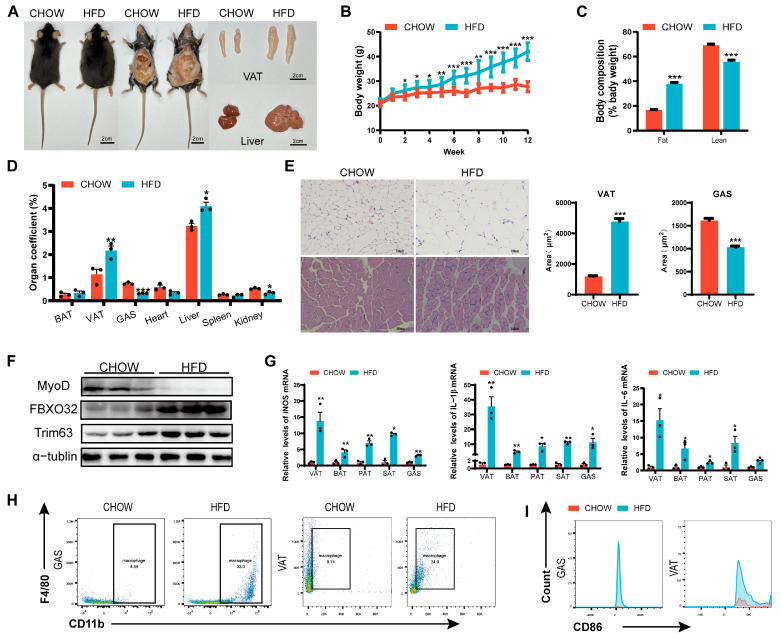
Skeletal muscle dysplasia in obese mice. (**A**) Representative images of CHOW diet (CHOW) and high-fat diet (HFD) mice, highlighting differences in body size, visceral adipose tissue (VAT), and liver morphology. (**B**) Body weight progression of CHOW and HFD mice over 12 weeks (weekly measurements) (*n* = 10). (**C**) Fat and lean mass composition in CHOW and HFD mice. (**D**) Organ coefficients (organ weight/body weight × 100%) (*n* = 10). (**E**) Histological sections (H&E staining) of VAT and GAS, bar = 100 μm. (**F**) The protein expression of MyoD and atrophy markers (FBXO32, and Trim63) in skeletal muscle (*n* = 3). (**G**) mRNA expression of inflammatory factors (*iNOS*, *IL-1β*, *IL-6*) in VAT and GAS (RT-PCR) (*n* = 3). (**H**) Flow cytometry analysis of CD11b+F4/80+ macrophage populations in skeletal muscle. (**I**) Proportion of M1-polarized macrophages (CD11b^+^F4/80^+^CD86^+^ cells) in skeletal muscle. * *p* < 0.05, ** *p* < 0.01, *** *p* < 0.001.

After 12 weeks of feeding, mice in the HFD group exhibited significant increases in body weight and body fat percentage (Figure 1A–C), accompanied by impaired glucose tolerance and insulin sensitivity (Figure S1A,B), indicating successful establishment of the obesity model. Postmortem examination revealed adipocyte hypertrophy (Figure 1E), marked visceral adipose tissue accumulation (Figure 1D), and marked hepatomegaly with pallor of the liver in HFD mice (Figure 1A), along with gastrocnemius muscle fiber atrophy and reduced muscle mass (Figure 1D,E). At the molecular level, skeletal muscle in HFD mice showed downregulation of MyoD and upregulation of FBXO32 and Trim63, confirming impaired muscle development (Figure 1F). Furthermore, elevated levels of pro-inflammatory cytokines (*iNOS*, *IL-1β*, *IL-6*) were observed in adipose and skeletal muscle tissues (Figure 1G), along with M1 macrophage (CD11b+F4/80+CD86+)-dominated infiltration (Figure 1H,I), demonstrating the increased levels of inflammation in the adipose and skeletal muscle tissue in obese mice.

### 3.2. HFD VAD-evs Accumulate in the Skeletal Muscle and Suppress Myogenesis

**Figure 2:**
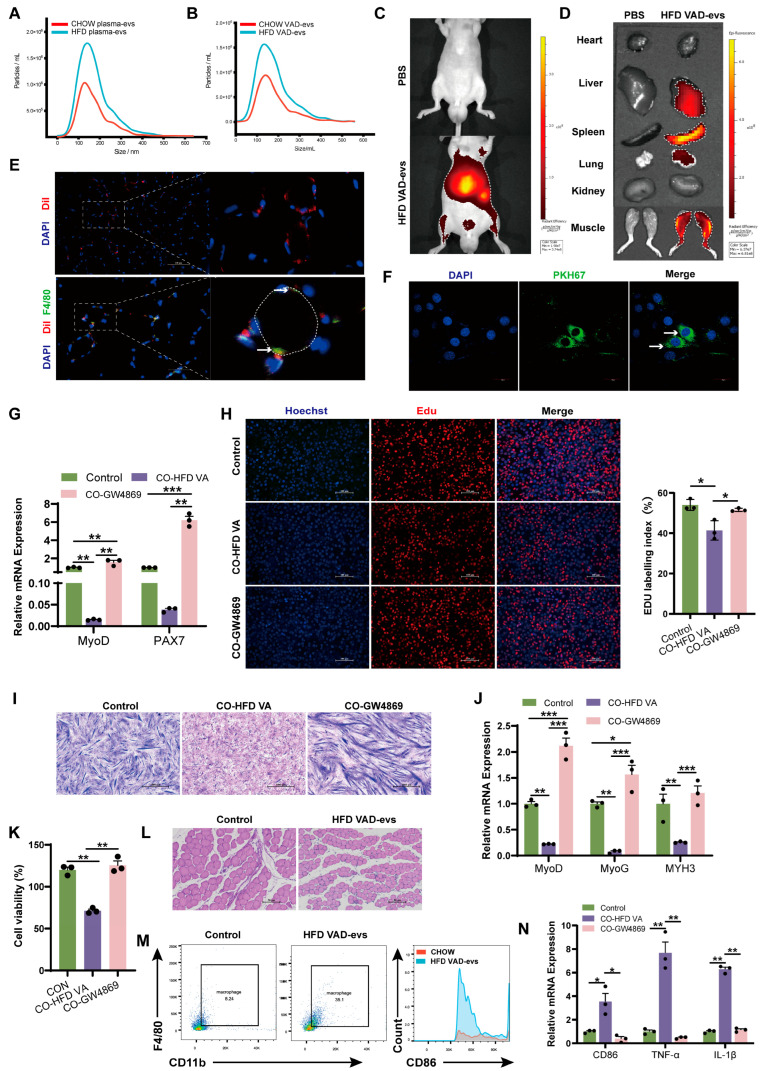
HFD VAD-evs accumulate in skeletal muscle and suppress myogenesis. (**A**,**B**) Nanoparticle tracking analysis (NTA) of plasma-derived EVs and visceral adipocyte-derived EVs (VAD-evs) from CHOW and HFD mice. (**C**,**D**) In vivo and in vitro fluorescence imaging of skeletal muscle 24 h after injection of DiR-labeled HFD VAD-evs into the tail vein. (**E**) Localization of DiL-labeled HFD VAD-evs (red) in skeletal muscle. Macrophages are F4/80 positive (green). Cell nuclei are stained with DAPI (blue). Colocalization of HFD VAD-evs with macrophages(white arrow). White dashed lines indicate myofibers. (**F**) Internalization of PKH67-labeled HFD VAD-evs (green) by C2C12 myoblasts (white arrows). (**G**) After treatment with GW4869 or PBS, HFD VAD-evs were co-cultured with C2C12 cells; the mRNA expression levels of the proliferation markers *MyoD* and *PAX7* of C2C12 cells were detected by RT-PCR (*n* = 3). (**H**) After treatment with GW4869 or PBS, HFD VAD-evs were co-cultured with C2C12 cells. Cell proliferation of C2C12 cells was detected by EDU staining. (**I**) After treatment with GW4869 or PBS, HFD VAD-evs were co-cultured with C2C12 cells. Cell differentiation of C2C12 cells was detected by Giemsa staining. (**J**) After being treated with GW4869 or PBS, HFD VAD-evs were co-cultured with C2C12 cells. mRNA expression levels of the differentiation markers *MyoD*, *MyoG*, and *MYH3* of C2C12 cells were detected by RT-PCR (*n* = 3). (**K**) After treatment with GW4869 or PBS, HFD VAD-evs were co-cultured with RAW264.7 cells. Cell viability of RAW264.7 cells was detected by CCK8 (*n* = 3). (**L**) H&E staining of skeletal muscle from CHOW mice injected with HFD VAD-evs, bar = 100 μm. (**M**) Flow cytometry analysis of CD11b+F4/80+ macrophages in the skeletal muscle of CHOW mice treated with HFD VAD-evs. (**N**) After treatment with GW4869 or PBS, HFD VAD-evs were co-cultured with RAW264.7 cells. mRNA expression levels of the inflammatory markers *CD86*, *TNF-α*, and *IL-1β* of RAW264.7 cells were detected by RT-PCR (*n* = 3). * *p <* 0.05, ** *p* < 0.01, *** *p* < 0.001.

Plasma-derived and visceral adipocyte-derived extracellular vesicles (VAD-evs) from HFD mice exhibited a characteristic cup-shaped morphology, with positive expression of the exosomal markers Alix and TSG101 but negative expression of Calnexin (Figure S2A,B). HFD VAD-evs displayed a higher particle yield, while HFD plasma-evs displayed a larger size distribution and elevated particle production (Figure 2A,B), suggesting dysregulated exosome secretion in obesity.

Following the fluorescent labeling of HFD VAD-evs, their in vivo biodistribution and accumulation were quantitatively assessed. Fluorescent tracer analysis demonstrated that HFD VAD-evs predominantly accumulated in the skeletal muscle of the hindlimbs, as well as in the liver, spleen, and lung (Figure 2C,D), suggesting a tissue-specific tropism toward the skeletal muscle. Immunofluorescence analysis of GAS tissue sections demonstrated the internalization of HFD VAD-evs by myoblasts (Figure 2E), while in vitro tracking confirmed that HFD VAD-evs were internalized by C2C12 cells (Figure 2F). These findings collectively demonstrated that myoblasts can receive cargo delivered by HFD VAD-evs, thus establishing the biological basis for the HFD VAD-ev-mediated modulation of myoblast cellular function. Co-cultures of C2C12 myoblasts with HFD VAD-evs resulted in the sustained downregulation of myoblast proliferation markers (*MyoD* and *PAX7*) (Figure 2G), reduced EDU-positive cell ratios (Figure 2H), and significant decreases in myotube number and fusion index (Figure 2I). Additionally, differentiation markers (*MyoD*, *MyoG*, and *MYH3*) of C2C12 cells were consistently suppressed (Figure 2J). After administering GW4869 (10 μM, 24 h, a potent neutral sphingomyelinase inhibitor that blocks extracellular vesicle secretion) to HFD visceral adipocytes, the phenomenon of HFD VAD-evs inhibiting the proliferation and differentiation of myoblasts was observed, demonstrating that HFD VAD-evs directly inhibit myogenesis.

Notably, HFD VAD-evs preferentially localized to macrophages within skeletal muscle (Figure 2E). In vivo, intravenous injection of HFD VAD-evs into CHOW mice increased macrophage infiltration in skeletal muscle and induced skeletal muscle atrophy (Figure 2L,M). In vitro, a co-culture of RAW264.7 macrophages with HFD VAD-evs suppressed macrophage proliferation (Figure 2K) and upregulated pro-inflammatory markers (*CD86*, *TNF-α*, and *IL-1β*) at the mRNA level (Figure 2N). These findings collectively indicate that HFD VAD-evs impair skeletal muscle development through dual regulatory mechanisms: (1) direct inhibition of the development of myoblasts and (2) indirect suppression of myogenesis via macrophage-mediated inflammation.

### 3.3. Inhibition of Exosome Secretion Ameliorates Obesity-Induced Skeletal Muscle Dysplasia

**Figure 3:**
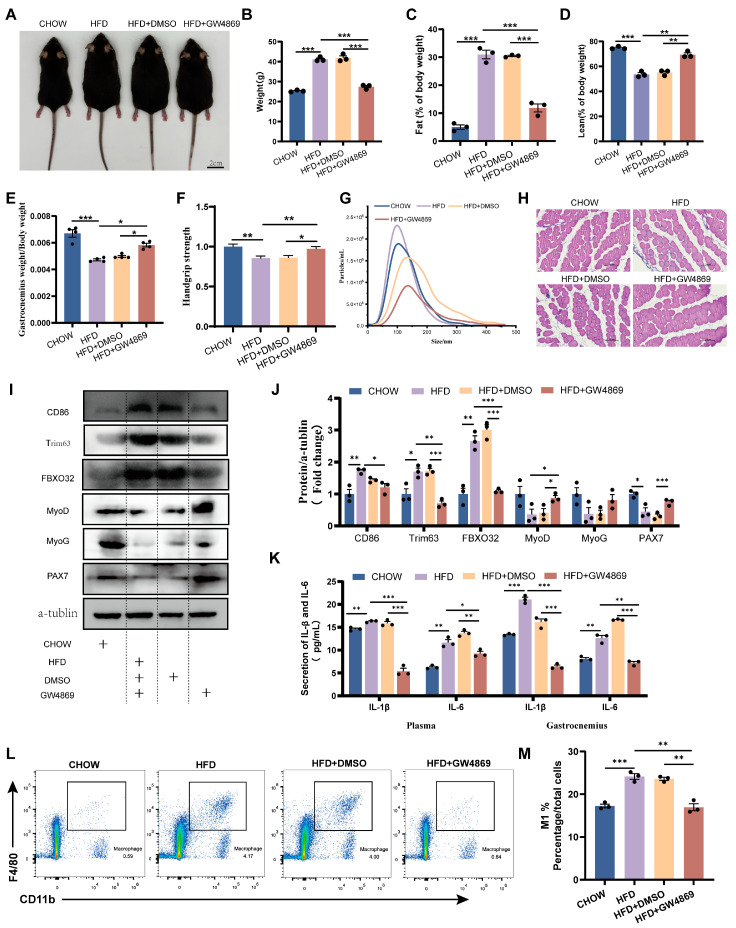
Inhibition of exosome secretion ameliorates obesity-induced skeletal muscle dysplasia. (**A**) Representative images of CHOW (CHOW diet mice), HFD (high-fat-diet mice), HFD+DMSO (HFD mice intraperitoneally injected with DMSO), and HFD+GW4869 mice (HFD mice intraperitoneally injected with GW4869). (**B**–**D**) Body weight, fat content, and muscle content of CHOW, HFD, HFD+DMSO, and HFD+GW4869 mice (*n* = 3). (**E**) Organ coefficient (gastrocnemius weight/body weight × 100%) of CHOW, HFD, HFD+DMSO, and HFD+GW4869 mice (*n* = 4). (**F**) Grip strength analysis of the forelimbs of CHOW, HFD, HFD+DMSO, and HFD+GW4869 mice (*n* = 10). (**G**) NTA of plasma-evs from CHOW, HFD, HFD+DMSO, and HFD+GW4869 mice. (**H**) Histological analysis of skeletal muscle from CHOW, HFD, HFD+DMSO, and HFD+GW4869 mice, bar = 100 μm. (**I**,**J**) Expression levels of CD86, Trim63, FBXO32, MyoD, MyoG, and PAX7 proteins in the skeletal muscle of CHOW, HFD, HFD+DMSO, and HFD+GW4869 mice (*n* = 3). (**K**) Detection of IL-1β and IL-6 levels in the plasma and skeletal muscle of CHOW, HFD, HFD+DMSO, and HFD+GW4869 mice using ELISA (*n* = 3). (**L**) Analysis of CD11b+F4/80+ macrophage clusters in skeletal muscle tissue of CHOW, HFD, HFD+DMSO, and HFD+GW4869 mice using flow cytometry. (**M**) Evaluation of the proportion of M1 (CD11b+F4/80+CD86+) macrophages in the skeletal muscle of CHOW, HFD, HFD+DMSO, and HFD+GW4869 mice. * *p* < 0.05, ** *p* < 0.01, *** *p* < 0.001.

In order to study the role of EVs in obesity-induced skeletal muscle dysplasia, GW4869 was used to pharmacologically inhibit exosome biogenesis in HFD mice. As shown in Figure 3A, GW4869 caused a noticeable reduction in the body size of HFD mice, but it was still larger than that of CHOW mice. Initial analysis revealed that compared to CHOW mice, HFD mice exhibited a significantly elevated body weight and fat mass fraction, coupled with a markedly depressed lean mass fraction, GAS mass fraction, and grip strength. GW4869 treatment can alleviate these changes in HFD mice (Figure 3A–F). Further analysis demonstrated that compared to HFD mice, the HFD+GW4869 group exhibited attenuated exosome secretion (Figure 3G). Compared to CHOW mice, HFD mice exhibited reduced skeletal muscle cross-sectional area and elevated protein expression of muscle atrophy markers (Trim63 and FBXO32), along with decreased trends in the myogenic regulators MyoD and MyoG and significantly reduced PAX7 levels (Figure 3H–J). Compared with the HFD mice, the cross-sectional area of skeletal muscle in the HFD + GW4869 mice increased, while the protein expression of Trim63 and FBXO32 significantly decreased, the protein expression of MyoD significantly increased, and the levels of MyoG and PAX7 showed an upward trend (Figure 3H–J). Additionally, compared with CHOW mice, HFD mice exhibited increased expression of pro-inflammatory cytokines (IL-1β, IL-6) in plasma and skeletal muscle (Figure 3K), elevated CD86 protein levels in skeletal muscle (Figure 3J), enhanced macrophage infiltration in skeletal muscle (Figure 3L), and increased M1 macrophage polarization in skeletal muscle (Figure 3M). GW4869 treatment reversed these alterations in HFD mice (Figure 3J–M). These findings confirm the mediating role of EV in obesity-associated skeletal muscle dysplasia and highlight that blocking exosome secretion alleviates myogenic impairment.

### 3.4. miR-155 Suppresses the Development of Myoblast

**Figure 4:**
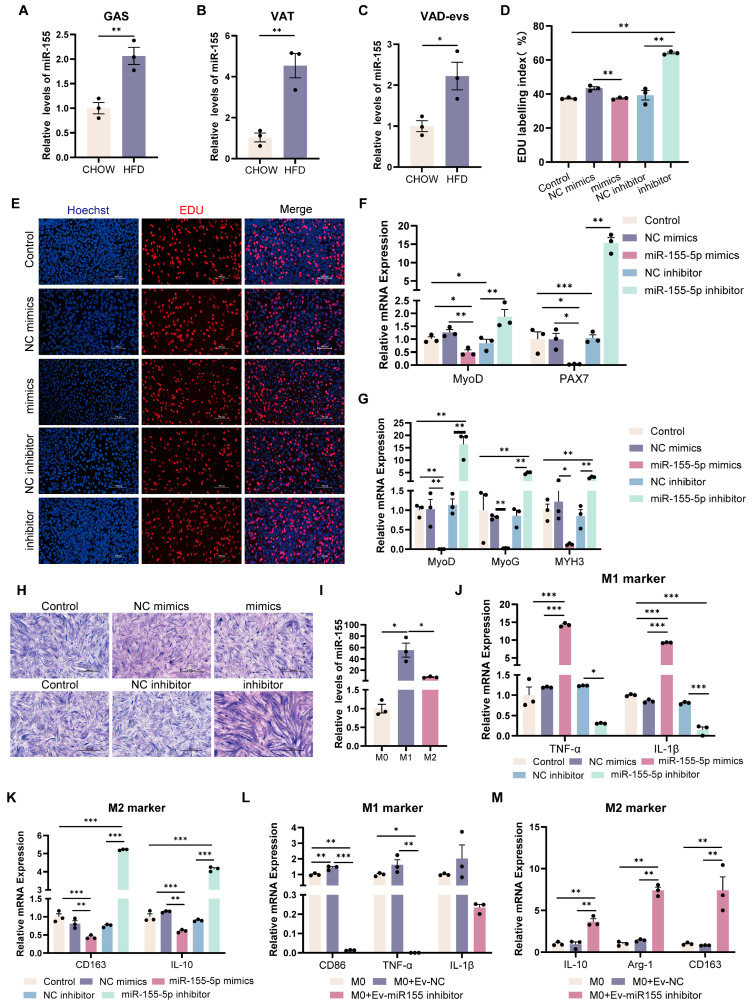
miR-155 suppresses the development of myoblasts and drives pro-inflammatory macrophage polarization. (**A**–**C**) miR-155 expression in gastrocnemius muscle (GAS), visceral adipose tissue (VAT), and visceral adipocyte-derived extracellular vesicles (VAD-evs) from CHOW vs. HFD mice (RT-PCR) (*n* = 3). (**D**,**E**) After C2C12 cells were transfected with miR-155 mimic, NC mimic, miR-155 inhibitor or NC inhibitor, the cell proliferation was detected by EDU staining. Blue represents nuclear staining (Hoechst) and red represents EDU staining, bar = 100 μm. (**F**) After C2C12 cells were transfected with miR-155 mimic, NC mimic, miR-155 inhibitor or NC inhibitor, the mRNA expression levels of the proliferation markers *MyoD* and *PAX7* were detected by RT-PCR (*n* = 3). (**G**) After myotube cells were transfected with miR-155 mimic, NC mimic, miR-155 inhibitor or NC inhibitor, the mRNA expression levels of the differentiation markers *MyoD*, *MyoG* and *MYH3* were detected by RT-PCR (*n* = 3). (**H**) After myotube cells were transfected with miR-155 mimic, NC mimic, miR-155 inhibitor or NC inhibitor, the cell differentiation was detected by means of Giemsa staining. (**I**) The expression levels of *miR-155* in different macrophage subtypes. RAW264.7 cells were treated with PBS, 20 ng/mL LPS (for M1 induction), or 20 ng/mL IL-4 (for M2 induction) (*n* = 3). (**J**) After RAW264.7 cells were transfected with miR-155 mimic, NC mimic, miR-155 inhibitor or NC inhibitor, the mRNA expression levels of M1 markers *TNF-α* and *IL-1β* were detected by RT-PCR (*n* = 3). (**K**) After RAW264.7 cells were transfected with miR-155 mimic, NC mimic, miR-155 inhibitor or NC inhibitor, the mRNA expression levels of M2 markers *CD163* and *IL-10* were detected by RT-PCR (*n* = 3). (**L**) The expression levels of M1 markers *CD86*, *TNF-α,* and *IL-1β* in RAW264.7 cells treated with HFD-VAD-evs and NC or miR-155 inhibitor (*n* = 3). (**M**) The expression levels of M2 markers *IL-10*, *Arg-1* and *CD163* in RAW264.7 cells treated with HFD-VAD-evs and NC or miR-155 inhibitor (*n* = 3). * *p* < 0.05, ** *p* < 0.01, *** *p* < 0.001.

To investigate the role of *miR-155* in obesity-induced skeletal muscle dysplasia, we analyzed miR-155 levels in HFD mice. Compared with CHOW mice, the relative levels of miR-155 were significantly upregulated in the skeletal muscle, adipose tissue, and VAD-evs of HFD mice (Figure 4A–C). These results suggest that miR-155 may mediate its effects via transport from the VAT to the skeletal muscle through VAD-evs. In order to verify our hypothesis, miR-155 was transfected into C2C12 cells. The results showed that compared with the NC mimic group, miR-155 mimics significantly reduced EDU incorporation in C2C12 cells (Figure 4D,E) and suppressed the expression of myoblast proliferation markers (*MyoD* and *PAX7*) (Figure 4F). Conversely, compared with the NC inhibitor group, miR-155-5p inhibitor increased EDU incorporation in C2C12 cells and enhanced the expression of *MyoD* and *PAX7* (Figure 4D–F). After the transfection of miR-155 into C2C12 cells followed by 3 days of differentiation, analysis revealed that compared with the NC mimic group, miR-155 mimics significantly downregulated key myogenic regulatory factors (*MyoD*, *MyoG*, and *MYH3*) and suppressed C2C12 cell differentiation (Figure 4G,H). Conversely, compared with the NC inhibitor group, miR-155-5p inhibitor significantly upregulated the relative expression levels of *MyoD*, *MyoG*, and *MYH3* and promoted C2C12 cell differentiation (Figure 4G,H). These results demonstrate that HFD VAD-evs suppress myoblast proliferation and differentiation via the exosomal transfer of miR-155.

### 3.5. miR-155-Depleted EVs Attenuate Pro-Inflammatory Macrophage Polarization

We assessed miR-155 levels in macrophages, observing that it was highly expressed in M1 but minimally expressed in M2 macrophages (Figure 4I). To investigate the role of miR-155 in macrophages, it was transfected into RAW264.7 cells. The results showed that compared with the NC mimic group, miR-155-5p mimics significantly promoted the expression of pro-inflammatory cytokines (*TNF-α* and *IL-1β*) while suppressing anti-inflammatory markers (*CD163* and *IL-10*) (Figure 4J,K). Conversely, compared with the NC inhibitor group, miR-155-5p inhibitor significantly reduced the expression of *TNF-α* and *IL-1β* while increasing *CD163* and *IL-10* (Figure 4J,K). Furthermore, compared with the group of macrophages with HFD VAD-evs encapsulated with a NC inhibitor (Ev-NC), the treatment of macrophages with HFD VAD-evs encapsulated with a miR-155 inhibitor (Ev-miR-155 inhibitor) markedly reduced *CD86*, *TNF-α*, and *IL-1β* expression and elevated *IL-10*, *Arg-1,* and *CD163* levels (Figure 4L,M). These results demonstrate that miR-155 drives pro-inflammatory macrophage polarization, whereas Ev-miR-155 inhibitor effectively counteracts this polarization.

### 3.6. miR-155 Inhibition Restores Skeletal Muscle Development and Reduces Inflammation in Obese Mice

**Figure 5:**
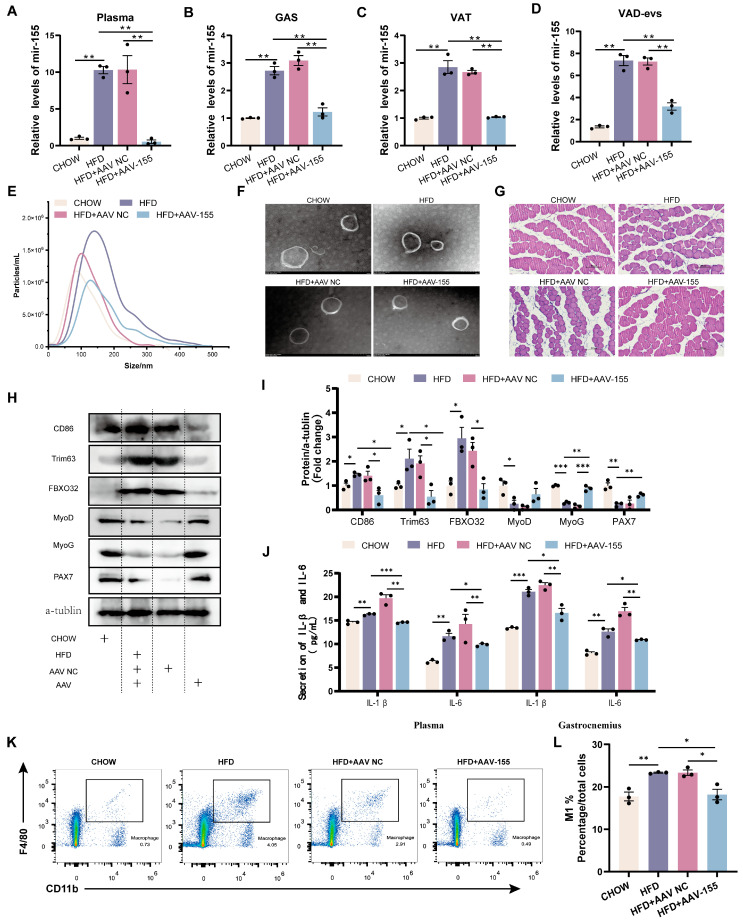
miR-155 inhibition restores skeletal muscle development and reduces inflammation in obese mice. (**A**–**D**) Expression levels of miR-155 in plasma, GAS, VAT, and VAD-evs of CHOW, HFD, HFD+AAV NC (HFD mice receiving intraperitoneal and intramuscular injections of AAV NC), and HFD+AAV-155 (HFD mice receiving intraperitoneal and intramuscular injections of AAV-155) (*n* = 3). (**E**) NTA of plasma-evs of CHOW, HFD, HFD+AAV NC, and HFD+AAV-155 mice. (**F**) TEM analysis of plasma-evs of CHOW, HFD, HFD+AAV NC, and HFD+AAV-155 mice. (**G**) Histological analysis of skeletal muscle of CHOW, HFD, HFD+AAV NC, and HFD+AAV-155 mice, bar = 100 μm. (**H**,**I**) Expression levels of CD86, Trim63, FBXO32, MyoD, MyoG, and PAX7 proteins in the skeletal muscle of CHOW, HFD, HFD+AAV NC, and HFD+AAV-155 mice (*n* = 3). (**J**) ELISA method for detecting the levels of IL-1β and IL-6 in plasma and skeletal muscle of CHOW, HFD, HFD+AAV NC, and HFD+AAV-155 mice (*n* = 3). (**K**) Flow cytometry analysis of CD11b+F4/80+ macrophage clusters in the skeletal muscle tissue of CHOW, HFD, HFD+AAV NC, and HFD+AAV-155 mice. (**L**) Evaluation of the proportion of M1 (CD11b+F4/80+CD86+) macrophages in the skeletal muscle of CHOW, HFD, HFD+AAV NC, and HFD+AAV-155 mice. * *p* < 0.05, ** *p* < 0.01, *** *p* < 0.001.

AAV mediated the knockdown of miR-155 in obese mice. Compared to HFD+AAV NC mice, HFD+AAV-155 mice displayed a significantly reduced relative expression of *miR-155* in the plasma (Figure 5A), skeletal muscle (Figure 5B), VAT (Figure 5C), and VAD-evs (Figure 5D), alongside decreased exosome particle yield in the plasma (Figure 5E). In addition, EVs from HFD+AAV-155 mice retained their characteristic cup-shaped morphology (Figure 5F).

Compared with CHOW mice, HFD mice exhibited a reduced skeletal muscle cross-sectional area, a decreased expression of myogenic proteins (MyoD, MyoG, and PAX7), and an elevated expression of atrophy markers (Trim63 and FBXO32) (Figure 5I). Relative to HFD+AAV NC mice, HFD+AAV-155 mice showed increased skeletal muscle cross-sectional area, significantly upregulated MyoG expression, a tendency toward increased MyoD and PAX7 expression, and reduced Trim63 and FBXO32 levels (Figure 5I). Notably, HFD mice displayed higher plasma and skeletal muscle levels of pro-inflammatory cytokines (IL-1β, IL-6) (Figure 5J), enhanced macrophage infiltration in skeletal muscle, an increased proportion of M1 macrophages, and a decreased proportion of M2 macrophages compared to CHOW mice (Figure 5K,L). Conversely, HFD+AAV-155 mice exhibited lower IL-1β/IL-6 levels in the plasma and skeletal muscle (Figure 5J), reduced macrophage infiltration, and a decreased M1 macrophage proportion compared to HFD+AAV NC mice (Figure 5K,L). These findings demonstrate that miR-155 suppression alleviates obesity-associated skeletal muscle dysplasia and inflammation.

### 3.7. HFD VAD-evs Impair Myogenesis via Macrophage-Dependent Mechanisms

**Figure 6:**
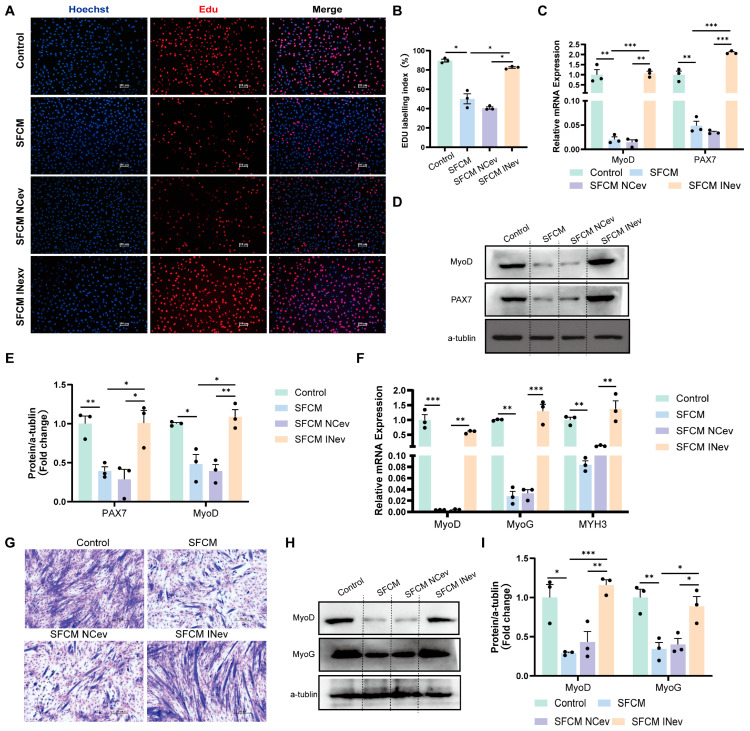
HFD VAD-evs impair myogenesis via macrophage-dependent mechanisms. (**A**,**B**) The incorporation of EDU in C2C12 cells in the control group (C2C12 cells were cultured with normal culture medium for 24 h), SFCM group (following 24 h of culture, serum-free conditioned medium was collected from RAW264.7 macrophages and utilized for the subsequent 24-h culture of C2C12 cells.), SFCM NCev group (following 24 h of culture, serum-free conditioned medium was collected from RAW264.7 cells treated with HFD VAD-evs loaded with the miR-155 inhibitor negative control and utilized for the subsequent 24-h culture of C2C12 cells.), and SFCM INev group (following 24 h of culture, serum-free conditioned medium was collected from RAW264.7 cells treated with HFD VAD-evs loaded with miR-155 inhibitor and utilized for the subsequent 24-h culture of C2C12 cells), with blue indicating nuclear staining (Hoechst) and red indicating EDU staining, bar = 100 μm (*n* = 3). (**C**) The mRNA expression of MyoD and PAX7 in the control group, SFCM group, SFCM NCev group, and SFCM INev group (*n* = 3). (**D**,**E**) The protein expression of MyoD and PAX7 in the control group, SFCM group, SFCM NCev group, and SFCM INev group (*n* = 3). (**F**) The expression levels of MyoD, MyoG, and MYH3 mRNA in myotube cells of the control group (C2C12 cells were cultured with differential medium for 3 days), SFCM group (serum-free conditioned medium was collected from RAW264.7 macrophages following 24 h of culture, supplemented with 2% horse serum, and subsequently utilized for 3-day differentiation induction of C2C12 cells.), SFCM NCev group (serum-free conditioned medium was collected from RAW264.7 macrophages treated with HFD VAD-evs loaded with the miR-155 inhibitor negative control following 24 h of culture, supplemented with 2% horse serum, and subsequently utilized for 3-day differentiation induction of C2C12 cells), and SFCM INev group (serum-free conditioned medium was collected from RAW264.7 macrophages treated with HFD VAD-evs loaded with miR-155 inhibitor following 24 h of culture, supplemented with 2% horse serum, and subsequently utilized for 3-day differentiation induction of C2C12 cells) (*n* = 3). (**G**) Giemsa staining in the control group, SFCM group, SFCM NCev group, and SFCM INev group. (**H**,**I**) The protein expression of MyoD and MyoG in the control group, SFCM group, SFCM NCev group, and SFCM INev group (*n* = 3). * *p* < 0.05, ** *p* < 0.01, *** *p* < 0.001.

Co-culture experiments with HFD VAD-evs/RAW264.7 cells/C2C12 cells demonstrated that, compared to the control group, the SFCM group exhibited reduced EDU incorporation in C2C12 cells (Figure 6A,B) and decreased protein expression and relative mRNA levels of myogenic markers (MyoD and PAX7) (Figure 6C–E). Relative to the SFCM NCev group, the SFCM INev group showed increased EDU incorporation (Figure 6A,B) and elevated protein expression and relative mRNA levels of MyoD and PAX7 (Figure 6C–E). These findings indicate that HFD VAD-evs carrying miR-155 suppress myoblast proliferation via a macrophage-dependent mechanism.

During post-co-culture differentiation (day 3), compared to the controls, the SFCM group displayed downregulated relative *MYH3* mRNA expression, reduced protein and mRNA expression of MyoD and MyoG, and diminished formation of multinucleated myotubes (Figure 6F–I). Conversely, the SFCM INev group exhibited upregulated relative *MYH3* mRNA expression, increased protein and mRNA expression of MyoD and MyoG, and enhanced myotube formation versus the SFCM NCev group (Figure 6F–I). This demonstrates that HFD VAD-evs carrying miR-155 inhibit myoblast differentiation through a macrophage-dependent mechanism.

Notably, Ev-miR-155 inhibitor effectively restored both proliferative and differentiative capacities in C2C12 cells. Collectively, these results establish that the conditioned medium from HFD VAD-ev-treated RAW264.7 macrophages suppresses both the proliferation and differentiation of C2C12 myoblasts, whereas blocking exosomal miR-155 effectively mitigates these inhibitory effects.

## 4. Discussion

Our findings reveal a dual mechanism underlying obesity-induced skeletal muscle dysplasia: the direct suppression of myoblast proliferation and differentiation by HFD VAD-evs carrying miR-155 and the indirect inhibition of myogenesis via miR-155 carried by HFD VAD-evs mediating the pro-inflammatory polarization of skeletal muscle macrophages. Critically, the inhibition of miR-155 in HFD VAD-evs reversed these pathological effects, highlighting its central role in the adipose–muscle crosstalk in obese individuals.

Clinical studies have established that obesity exacerbates skeletal muscle inflammation and metabolic dysregulation, leading to reduced muscle mass and function [40]. Consistent with this, our HFD mice exhibited increased visceral adiposity and skeletal muscle atrophy, confirming obesity-associated skeletal muscle dysplasia (Figure 1).

In obese individuals, visceral adipose tissue contributes to skeletal muscle mass reduction by impairing muscle protein synthesis [9], while the regulatory role of exosomes as adipocyte-derived signaling mediators in biological processes has been established [41]. Our results demonstrated that HFD VAD-evs predominantly accumulated in the hindlimb skeletal muscle in vivo (Figure 2C,D), leading to skeletal muscle dysplasia, while blocking exosome secretion reversed this phenotype (Figure 2 and Figure 3). In vitro, co-cultures of C2C12 myoblasts with HFD VAD-evs attenuated myogenesis, which was alleviated upon suppression of exosome release (Figure 2G–J). These data confirm the direct inhibitory effect of VAD-evs on skeletal muscle development. Interestingly, in vivo observations revealed increased macrophage infiltration and elevated inflammatory cytokine levels in the skeletal muscle of obese mice (Figure 1H,I). In vitro, co-cultures of RAW264.7 cells with HFD VAD-evs suppressed macrophage proliferation and heightened inflammatory responses, effects that were mitigated when HFD VAD-ev secretion was inhibited (Figure 2K,N). These findings suggest that HFD VAD-evs harbor pro-inflammatory factors that drive macrophage-associated inflammation both in vivo and in vitro.

MiRNAs regulate diverse biological processes in multiple organs and tissues, including skeletal muscle mass maintenance, fiber-type specification, and muscle pathology [42,43]. Several miRNAs, such as miR-155 [44], miR-146a [45], miR-132 [46], miR-21 [47], miR-26 [48], miR-29 [49], miR-181a [50], miR-145 [51], miR-223 [52], and miR-122 [53], have been widely implicated in inflammatory pathways. A subset of 11 inflammation-related miRNAs were analyzed via qPCR profiling, revealing that miR-155 exhibited the most pronounced upregulation in HFD VAD-evs [52]. miR-155 is abundantly present in exosomes derived from obese adipose tissue, and its pro-inflammatory role has previously been reported [54,55,56]. In this study, obesity significantly increased miR-155 levels in adipose tissue, plasma extracellular vesicles, VAD-evs, and skeletal muscle (Figure 4A–D), consistent with observations in human obese adipose tissue [22]. These findings imply that miR-155 may be transferred to the skeletal muscle via HFD VAD-evs. In vitro, miR-155 overexpression suppressed C2C12 myoblast development (Figure 4D–H) and promoted the pro-inflammatory polarization of RAW264.7 macrophages (Figure 4J,K), while miR-155 inhibition reversed these effects. In vivo, miR-155 knockdown alleviated obesity-induced skeletal muscle dysplasia, macrophage infiltration, and muscle inflammation (Figure 5G–I). Studies have established that adipose tissue macrophages can secrete exosomes containing miR-155 that are delivered to the muscle, liver, and adipocytes [30]. Collectively, our data demonstrate that the HFD VAD-ev-mediated delivery of miR-155 contributes to skeletal muscle dysplasia and inflammation in obese mice.

HFD VAD-evs predominantly accumulate in macrophages rather than myocytes within skeletal muscle, as demonstrated by our findings. The distinct effects of HFD VAD-evs on macrophages and myocytes were experimentally validated (Figure 2G–N). Given the spatial proximity between myocytes and skeletal muscle macrophages, as well as the enrichment of HFD VAD-evs in this niche, we established a co-culture system of HFD VAD-evs/RAW264.7 cells/C2C12 cells to mimic their in vivo interactions. This system preserved macrophage-derived cytokines while minimizing direct exosomal effects on myoblasts. The results showed that conditioned medium from HFD VAD-ev-treated RAW264.7 cells suppressed C2C12 myogenesis, while Ev-miR-155 inhibitor alleviated this suppression (Figure 6). These observations indicate that HFD VAD-evs indirectly impair skeletal muscle development via the miR-155-mediated modulation of macrophages. Prior studies highlight the critical role of macrophage pro-/anti-inflammatory polarization in skeletal muscle maintenance and regeneration, with inflammatory cytokines implicated in myofiber atrophy, myogenic suppression, and functional impairment [57]. For instance, IL-6 has been reported to induce skeletal muscle atrophy [58], while TNF-α released by macrophages promotes muscle pyroptosis [59]. We hypothesize that miR-155 in HFD VAD-evs drives macrophage polarization toward a pro-inflammatory phenotype, leading to cytokine secretion that inhibits myoblast proliferation and differentiation. However, the precise mechanisms underlying this regulatory axis require further investigation.

While our study focused on miR-155 as a paracrine mediator secreted by visceral adipocyte-derived EVs (VAD-evs) to inhibit myogenesis, miR-155 has also been established to intrinsically regulate adipocyte function. Specifically, obesity-associated inflammation induces miR-155 expression in adipose tissue, where it promotes inflammatory responses, chemokine production, and macrophage infiltration [22]. Extensive evidence supports bidirectional communication between the adipose tissue and skeletal muscle, mediated by adipokines, myokines, and exosomes [60,61]. The skeletal muscle secretes myokines (e.g., irisin, IL-6) that modulate adipose tissue metabolism [22,62]. Although our data demonstrate that visceral adipose tissue (VAT) impairs skeletal muscle development via EV-mediated miR-155 delivery, the reciprocal influence of skeletal muscle on VAT remains unexplored in this model. Future studies should therefore address whether myoblast-derived factors regulate miR-155 expression in adipocytes and if muscle atrophy exacerbates VAT inflammation through feedback loops.

In summary, obesity-associated VAD-evs deliver miR-155 to the skeletal muscle, where it disrupts myogenesis through dual pathways: the direct suppression of myoblast development and the indirect inhibition of myoblast proliferation and differentiation by exacerbating macrophage-driven inflammation. Targeting miR-155 in HFD VAD-evs represents a promising strategy to mitigate obesity-induced skeletal muscle dysfunction.

## Figures and Tables

**Table 1:** Grouping and treatment of CHOW-fed Mice.

Group	Treatment
CHOW group	
PBS group	PBS was injected into the tail vein on day 1, day 3, day 5, and day 7 (starting at 6 weeks of age).
HFD VAD-ev group	HFD VAD-evs were injected into the tail vein on day 1, day 3, day 5, and day 7 (5.0 × 10^10^ particles/mouse, starting at 6 weeks of age).

**Table 2:** Grouping and treatment of HFD-fed mice.

Group	Treatment
HFD group	Intraperitoneal injection of PBS (every day, 9th–16th week of age).
HFD+DMSO group	Intraperitoneal injection of DMSO (every day, 9th–16th week).
HFD+GW4869 group	Intraperitoneal injection of GW4869 (0.5 mg/kg body weight/day, every day, 9th–16th week of age).
HFD+AAV NC group	AAV NC was injected intraperitoneally on the first day and via the tail vein on the third day, respectively (100 μL/day, starting at 9 weeks of age).
HFD+AAV-155 group	AAV-155 was injected intraperitoneally on the first day and via the tail vein on the third day, respectively (100 μL/day, starting at 9 weeks of age).

**Table 3:** Primers used to assess gene structure.

Gene	Primer Sequece
miR-155-5p Forward	GCGCGTTAATGCTAATTGTGAT
miR-155-5p Reverse	AGTGCAGGGTCCGAGGTATT
miR-155-5p-loop	GTCGTATCCAGTGCAGGGTCCG AGGTATTCGCACTGGATACGACACCCCT
U6 Forward	TCGCTTCGGCAGCACA
U6 Reverse	AACGCTTCACGAATTTGCGT
β-actin Forward	TTGCTGACAGGATGCAGAAG
β-actin Reverse	ACATCTGCTGGAAGGTGGAC
iNOS Forward	GCGCTCTAGTGAAGCAAAGC
iNOS Reverse	AGTGAAATCCGATGTGGCCT
IL-1β Forward	TGCCACCTTTTGACAGTGATG
IL-1β Reverse	TTCTTGTGACCCTGAGCGAC
IL-6 Forward IL-6 Reverse	GGAAATCGTGGAAATGAG CCAGAAGACCAGAGGAAA
MyoD Forward	GTAGCAAGATCCACTCACCCT
MyoD Reverse	CTGGAGCCATTTGGCAGTAGT
MyoG Forward	AACTACCTTCCTGTCCACCTTC
MyoG Reverse	CACAGACTTCCTCTTACACACCT
MYH3 Forward	TCCAAACCGTCTCTGCACTGTT
MYH3 Reverse	AGCGTACAAAGTGTGGGTGTGT
CD86 Forward	GCAGCACGGACTTGAACAAC
CD86 Reverse	CCTTTGTAAATGGGCACGGC
TNF-α Forward TNF-α Reverse	ACCGTCAGCCGATTTGCTAT TTGGGCAGATTGACCTCAGC
IL10 Forward	CCAAGGTGTCTACAAGGCCA
IL10 Reverse	GCTCTGTCTAGGTCCTGGAGT
Arg1 Forward	CGGCAGTGGCTTTAACCTTG
Arg1 Reverse	TTGGGAGGAGAAGGCGTTTG
CD163 Forward	TGGGTGGGGAAAGCATAACT
CD163 Reverse	AAGTTGTCGTCACACACCGT

## Data Availability

No new data was generated.

## Supplementary Materials

The following supporting information can be downloaded at: https://www.mdpi.com/article/10.3390/cells14171302/s1.

## Abbreviations

CHOW	CHOW diet
HFD	High-fat diet
VAT	Visceral adipose tissue
VA	Visceral adipocyte
BAT	Brown adipose tissue
PAT	Perivascular adipose tissue
SAT	Subcutaneous adipose tissue
GAS	Gastrocnemius
OGTT	Oral glucose tolerance test (OGTT)
ITT	Insulin tolerance test
NTA	Nanoparticle tracking analysis
TEM	Transmission electron microscopy
HFD VA	High-fat diet visceral adipocyte
HFD VAD-ev	High-fat diet visceral adipocyte -derived extracellular vesicle
CO-HFD VA	C2C12 cells were seeded in the lower chamber of Transwell plates, while HFD VA cells were plated in the upper chamber
CO-GW4869	C2C12 cells were seeded in the lower chamber of Transwell plates, while HFD VA cells were plated in the upper chamber with GW4869
HFD+DMSO	HFD mice were intraperitoneally injected with DMSO
HFD+GW4869	HFD mice were intraperitoneally injected with GW4869
F4/80	Mouse egf-like module-containing mucin-like hormone receptor-like 1
CD11b	Cyclin-dependent kinase 11b
CD86	T-lymphocyte activation antigen CD86
CD206	Macrophage mannose receptor 1
MyoD	Myoblast determination protein
PAX7	Paired box protein PAX-7
MyoG	Myogenic
MYH3	Myosin heavy chain 3
FBXO32	Recombinant f-box protein 32
Trim63	Tripartite motif-containing protein 63
CCL2	C C motif ligand 2
IL-6	Interleukin 6
IL-1β	Interleukin 1β
iNOS	Nstitute nacional de obras sanitarias
TNFa	Tumor necrosis factor-a
M0+Ev-NC	RAW264.7 cells treated for 24 h with HFD VAD-evs loaded with the miR-155 inhibitor negative control.
M0+Ev-miR155 inhibitor	RAW264.7 cells treated for 24 h with HFD VAD-evs loaded with miR-155 inhibitor.
AAV-NC	AAV vector treatment was used as the negative control
HFD+AAV NC	AAV NC was injected intraperitoneally on the first day and via the tail vein on the third day, respectively (100 μL/day, start at 9 weeks of age).
HFD+AAV-155	AAV-155 was injected intraperitoneally on the first day and via the tail vein on the third day, respectively (100 μL/day, start at 9 weeks of age).
SFCM	SFCM was harvested from RAW264.7 cells treated for 24 h with HFD VAD-evs
SFCM NCev	SFCM was harvested from RAW264.7 cells treated for 24 h with HFD VAD-evs loaded with the miR-155 inhibitor negative control.
SFCM INev	SFCM was harvested from RAW264.7 cells treated for 24 h with HFD VAD-evs loaded with miR-155 inhibitor.
