# Supplementary figures and images for: Extracellular Vesicle-Mediated miR-155 from Visceral Adipocytes Induces Skeletal Muscle Dysplasia in Obesity
Source: Cells. 2025 Aug 22;14(17):1302. doi: 10.3390/cells14171302 (PMC12427855; doi:10.3390/cells14171302)

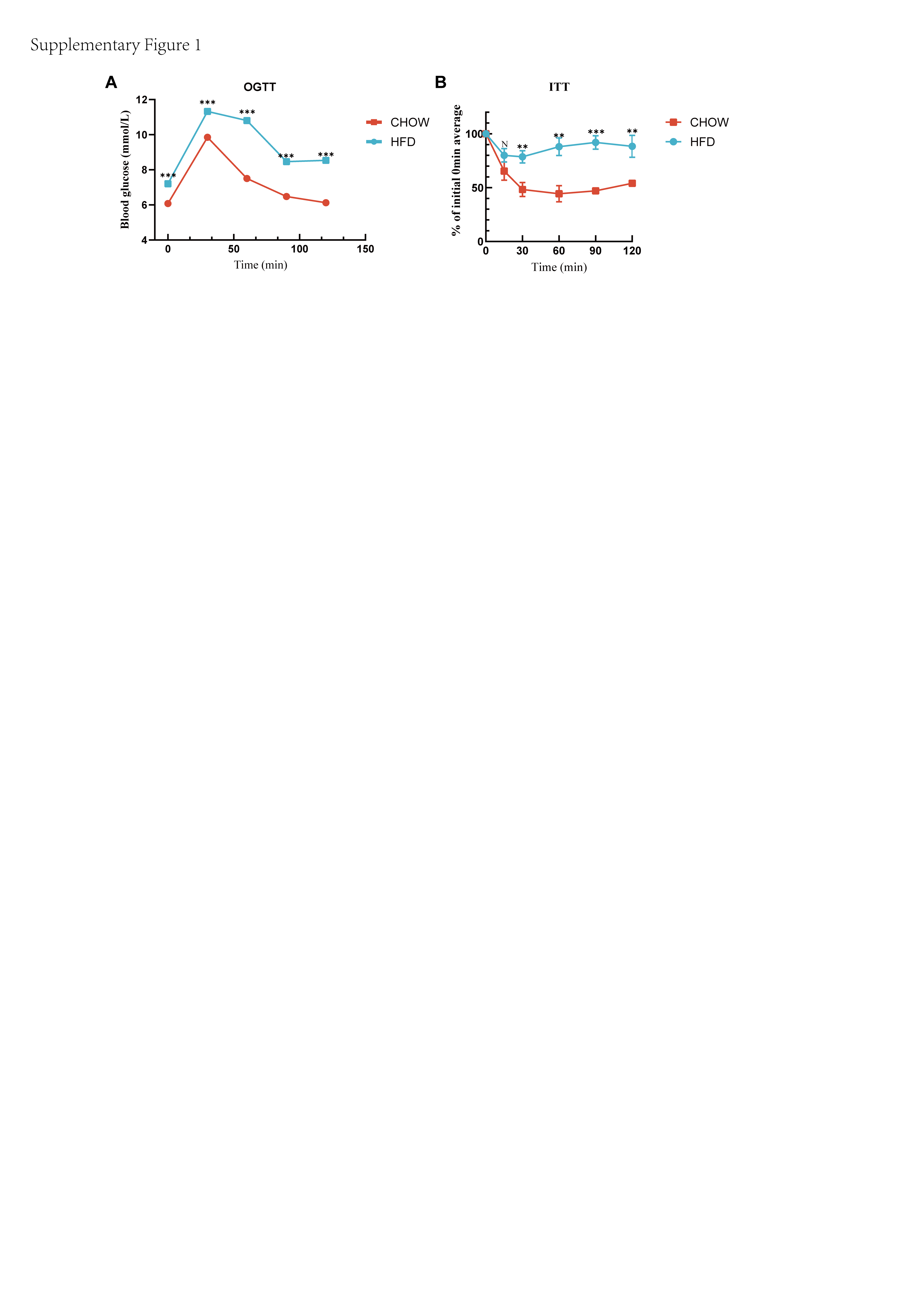

Supplement: Supplementary file 1 [file cells-14-01302-s001.zip › Supplementary Figure 1.tif]

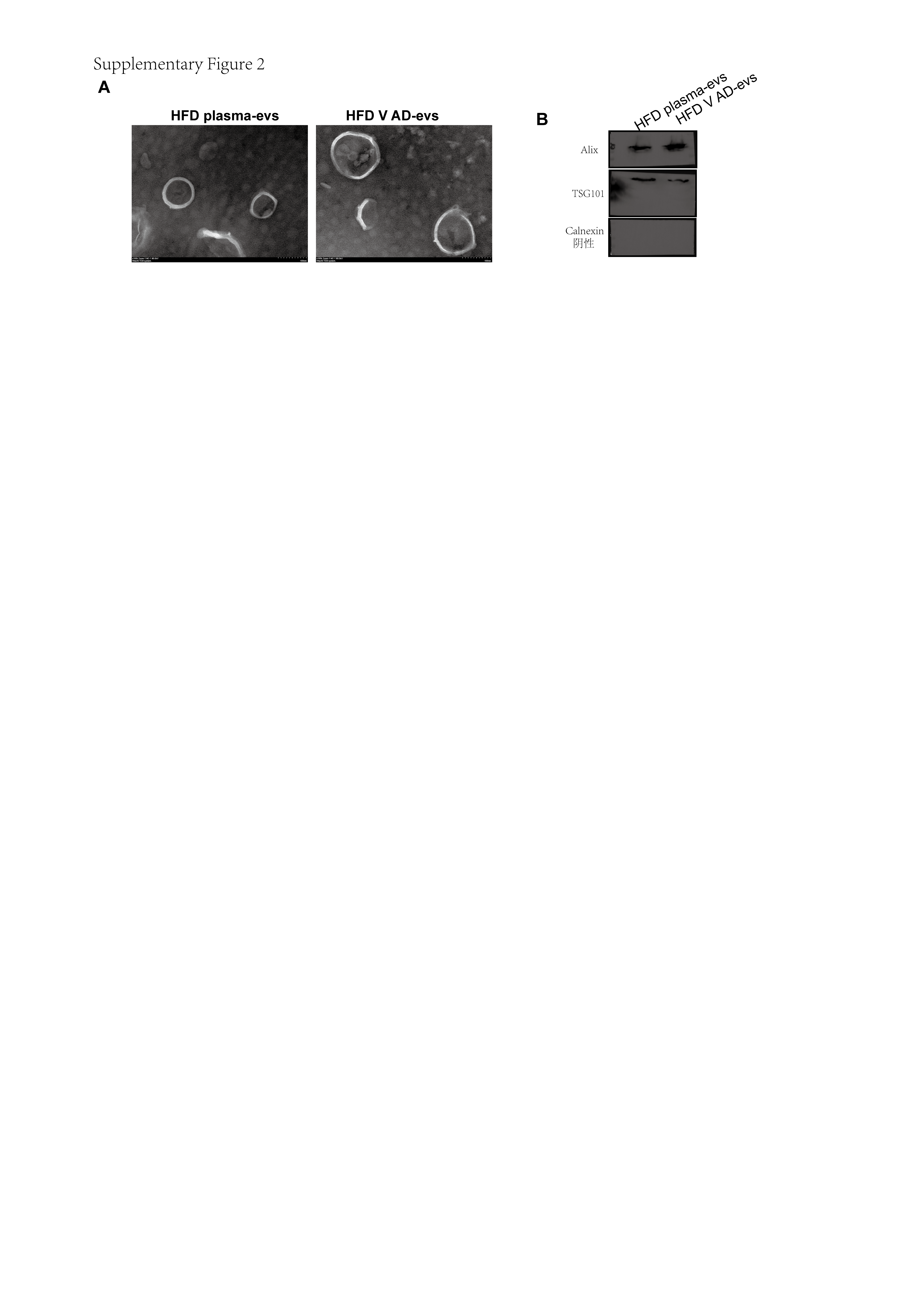

Supplement: Supplementary file 1 [file cells-14-01302-s001.zip › Supplementary Figure 2.tif]
